# Facial Attractiveness of Chinese College Students With Different Sexual Orientation and Sex Roles

**DOI:** 10.3389/fnhum.2019.00132

**Published:** 2019-04-30

**Authors:** Juan Hou, Lumeng Sui, Xinxin Jiang, Chengyang Han, Qiang Chen

**Affiliations:** ^1^Department of Philosophy, Anhui University, Hefei, China; ^2^College of Education, Zhejiang University, Hangzhou, China

**Keywords:** facial attractiveness, sexual dimorphism, homosexuality, sex role, eye tracking

## Abstract

Facial attractiveness refers to a positive and joyful emotional experience induced by the face of a target person and the extent to which other people are driven to be close to their wishes. Since the 1970s, face attractiveness has gradually emerged in western psychological research, but most of the studies were confined to heterosexuals. More recently, some scholars have pointed out that sexual orientation may affect the judgment of facial attractiveness of individuals. Based on previous literature, this study proposed to explore the different facial attractiveness of individuals with different sexual orientations and sexual roles. Participants in this study were divided into two types (according to sexual orientation and sexual role) by the Sex Role Inventory for College Students (CSRI). Also, the eye-tracking technique was used to record the path of eye movements, where face images were manipulated by sexual dimorphism clues. The results showed that (1) compared to heterosexual men, homosexual men were significantly more likely to choose masculine faces as more attractive faces in paired faces; (2) male homosexuals are likely to have the feminization bias, and female homosexuals are likely to have the masculinization bias; and (3) the masculine faces are more attractive than feminine faces to participants whose sex role is feminine type and androgynous type.

## Introduction

Facial attractiveness refers to the greatest degree of pleasure given to the senses (Li and Chen, 2010). Previous studies have investigated that faces were identified by averageness, symmetry, and sexual dimorphism. All of the three elements are regarded to contribute to the attractiveness of an individual’s face (Grammer and Thornhill, 1994; Perrett et al., 1999; Rhodes, 2006; Rennels et al., 2008). Also, studies relying on attractiveness assessments of static facial images are ecologically valid (Kościński, 2013).

However, what are the factors that would influence people’s judgments of facial attractiveness? Chen et al. (1997) classified the factors affecting facial attractiveness into two hypotheses: the observer hypothesis and the owner hypothesis.

The observer hypothesis refers to the observer’s characteristics (such as the observer’s physiological, cognitive, and sociocultural factors), which play an important role when judging facial attractiveness (Kou et al., 2013). For example, according to Zhang and Zheng (2016), the degree of angled faces (angle effect) is an essential factor in the assessment of facial attractiveness. Their results indicated that vertical faces were more attractive than other faces and that left-leaning faces were more attractive than right-leaning faces.

Nevertheless, the owner hypothesis focuses on features inherent in the physiognomy of the owner’s face, which would affect their judgment of face attractiveness (Little and Perrett, 2002). Hence, this hypothesis believes that face attractiveness is a stable trait of people (Jones et al., 2004). The research mainly uses the facial metric method to measure the faces. The primary method is to quantify every landmark point of a face by using Morph to change the position, distance, arrangement, and proportion of the landmark points, determining the geometric characteristics of the face. This method affects the judgment of facial attractiveness, such as averageness, symmetry, and sexual dimorphism (Kou et al., 2013).

Among them, sexual dimorphism refers to mature men and women after the development of adolescence; their secondary sexual characteristics gradually develop the body of sexual dimorphism, that is, masculine and feminine (Perrett et al., 1998). Sexual dimorphism not only is an essential indicator of facial attractiveness but also plays an important role in mate selection (Wen and Zuo, 2012). More precisely, according to Conroy-Beam et al. (2015), sexual dimorphism in mate selection “has cascading sex-specific consequences for important human endeavors such as marriage, child rearing, and divorce, all which suggest that sexes face are importantly different evolutionary histories and trajectories.”

Studies on sexual dimorphism have found that feminized female faces are considered attractive (Rhodes, 2006). However, there is no consistent conclusion in preference for male faces. Some researchers have found that women prefer masculine male faces (Miller and Todd, 1998; Gangestad and Scheyd, 2005). However, other studies argued that women have a weak preference for masculinized male faces, but a stronger preference for feminized male faces (Rhodes et al., 2003; DeBruine et al., 2006; Welling et al., 2007; Little et al., 2008). Wen and Zuo (2012) evaluated women’s judgments of the attractiveness of men’s faces under the condition of sexual dimorphism and found that female participants preferred masculinized male faces. Also, the mean pupil dilation and the mean fixation count on male faces were significantly higher than that on female faces. Yang (2015) had used synthetic face images as his experimental materials and adopted eye-track technology as well as questionnaires to explore male and female preferences. They observed that compared with androgynous faces, both male and female participants preferred masculine male faces. Meanwhile, the eye movement data showed that although longer gaze duration and a greater number of fixations were found when males and females were watching masculine male faces, no significant differences appeared. Moreover, in their study, they also discovered that compared to androgynous faces, the behavior data showed that both males and females prefer feminine female faces. Besides, they noted that when viewing feminine female faces, males, and females had a longer fixation duration and a greater number of fixations.

Nonetheless, previous studies mostly focused on sexual dimorphism in heterosexual groups and seldom considered different sexual orientation. Also, as the number of homosexuals increases, some researchers have pointed out that sexual orientation may affect the individual’s judgment of facial attractiveness (Steffens et al., 2013). For heterosexual groups, attractive opposite-sex faces are more rewarding, while homosexual groups think attractive same-sex faces are more satisfying (Kranz and Ishai, 2006). Glassenberg et al. (2010) used four types of face pictures (masculinized and feminized male and female faces) to examine the preference of different sexual orientations for masculinity–femininity. They noted that homosexual men had stronger preferences for masculinized male faces, whereas homosexual women had stronger preferences for masculinized female faces than heterosexual women. Other research studies noticed that homosexual men were able to identify more male faces than female faces, whereas heterosexual men can recognize more female faces than male faces (Beres et al., 2014; Li, 2016, unpublished). These findings further confirmed the importance of sexual orientation in the field of facial attractiveness.

Through prior studies on sexual dimorphism and sexual orientation, we know that differences in sex and sexual orientation would influence individuals’ face preferences. But what about individuals’ psychological sex differences? Does an individual’s psychological awareness of their gender affect their face preferences?

Qian et al. (2000) compiled the Sex Role Inventory for College Students (CSRI), which divides college students into four sex roles: masculine, feminine, androgynous, and undifferentiated, accounting for 24.7, 15.4, 31.5, and 28.4% of males and 22.5, 28.0, 25.0, and 24.5% of females, respectively. However, her study did not divide participants according to sexual orientation; therefore, when the participants were divided into different sexual orientation groups, would the proportion of their sex roles be different? Do the different sex role types affect their face preferences? These questions are worth studying.

According to previous studies, there is no consensus among researchers on face preferences of homosexuals. Bailey et al. (1997) have suggested that homosexual men prefer masculine male faces, while homosexual women have no preference for either masculinity or femininity in women. Echoing to this, Glassenberg et al. (2010) discovered that homosexual males showed stronger preferences for masculinity in male faces than did all of the other groups (homosexual women and heterosexual men and women). Also, homosexual women demonstrated stronger preferences for masculinity in female faces than did heterosexual women. Therefore, the first hypothesis we examined was that homosexual men might prefer masculinized faces, while homosexual women have no significant preference. Turning to the question of the relationship between participants and their sex roles, Qian et al. (2000) found that the sex roles of most men were the androgynous type and that for women were the feminine type. Thus, we speculated that the proportion of sex roles in our study would change, and we hypothesized that most homosexual men sex roles would be a feminine type and most homosexual women sex roles would be a masculine type. Finally, we also want to observe if a particular connection exists between participants’ sex role and their facial preference. In the Johnston et al. (2001) study, they found that different Bem Sex Role Inventory (BSRI) sex role groups exhibit different face preferences. Combined with prior hypotheses of this study, therefore, we hypothesized that participants with masculine sex roles preferred feminized faces, while participants with feminine sex roles preferred masculinized faces.

Besides, we found that previous studies mostly invited participants to evaluate the paired stimuli subjectively; for example, Sun et al. (2012) used forced choice to investigate the influence of the position of the left-side and the right-side face to facial attractiveness. However, other studies argue that the eye-tracking technique is a more efficient way to collect data on participants’ attention, which could shed light on the relative traits when people are making attractiveness judgments. Eye tracking is a method based on using computer equipment to record eye movements and the corresponding pupillary–corneal reflection (Richards et al., 2015). In the Corbetta et al. (1998) study, they pointed out that the brain captures information throughout the fixations of eye movement. Meanwhile, through recording the duration and the location of fixations of eye movement, it is conceivable to learn what characteristics a participant would consider most relevant. If the participant has an interest in a feature, his or her eyes will be attracted to this specific feature (Berlyne, 1958). Furthermore, in eye-tracking experiments, regardless of participants’ attention, whether it be endogenous or exogenous, the eye-tracking technique can quickly capture and transfer results to an intuitionistic data to achieve a goal (Ruz and Lupiáñez, 2002; Dixson et al., 2011). For example, Zhang et al. (2016) used the eye-tracking technique to explore the effect of smiling on the cognitive processing of facial attractiveness. The results showed that smiling influences face attractiveness. The mouth and the eyes are crucial for individuals’ judgment of facial attractiveness. As a new and fundamental cognitive method, the eye-tracking technique can provide immediate and objective eye movement indicators for cognitive processing. In this study, the eye-movement technique was used to discover the unintentional attention of participants with different sexual orientations when they were viewing different kinds of faces. Meanwhile, adopting questionnaires and the combination of subjective and objective methods could give us a more precise and more comprehensive understanding of data.

In conclusion, on the basis of previous studies to explore whether there are differences in their face preferences for sexual dimorphism clues, the current study proposes to use the CSRI and Kinsey Scale to classify participants into two types: sexual orientation and sex roles. From a more objective point of view, this study was undertaken to provide unbiased eye movement indicators for sexual dimorphism on the impact of facial attractiveness. Meanwhile, in this study, we examine the following hypotheses on facial attractiveness, which arise from these considerations: (a) homosexual men might prefer masculinized faces, while homosexual women have no significant preference; (b) most homosexual men’s sex roles would be a feminine type and most homosexual women’s sex roles would be a masculine type; and (c) participants’ sex roles in masculine type prefer feminized faces, while participants with feminine sex role type prefer masculinized faces.

## Materials and Methods

### Ethics Statement

The study was approved by the Human Research Ethics Committee of Anhui University. All participants gave consent to participate in the study and principles expressed in the Declaration of Helsinki were strictly followed. Participants were undergraduate students. Informed consent was obtained in written form from all participants.

The youngest participant was 18 years old. We did not obtain informed consent from the next of kin, caretakers, or guardians on behalf of the minors/children enrolled in our study. These college students were considered to have comparable intelligence and ability, and able to take charge of their behaviors.

### Participants

A total of 95 participants came from a number of online sources (including Chinese homosexual app, Tencent homosexual online groups, QQ, and WeChat) where we posted advertisements asking for men and women who were interested in helping with a 40-min eye-tracking study on facial attractiveness (mean age = 20.06, *SD* = 1.47). All participants were between the ages of 18 and 24 years. Of them, 22 men and 23 women identified themselves as heterosexual, and 25 men and 25 women identified themselves as homosexual. Sexual orientation was determined by asking participants to select one of seven statements that best described their sexual orientation. The eight statements provided in the survey were taken from the Kinsey scale (Kinsey et al., 1949). All participants were right-handed and had normal or corrected-to-normal vision. Before the experiment, participants were informed about the study’s purpose and procedure, and they were paid 30 RMB after the experiment.

To separate our participants into four groups (homosexual and heterosexual male and female), individuals who rated themselves as “exclusively homosexual,” “predominantly homosexual, only incidentally heterosexual,” and “predominantly homosexual, but more than incidentally heterosexual” were classified as homosexual, whereas individuals who rated themselves as “exclusively heterosexual,” “predominantly heterosexual, only incidentally homosexual,” and “predominantly heterosexual, but more than incidentally homosexual” were classified as heterosexual. Bisexual people were not the focus of this study, so they were not brought into our data analysis.

### Questionnaire Measures

#### The Kinsey Scale

The Kinsey Scale only has one item, which is used as a criterion for judging the sexual orientation of the participants. The scale is rated on an eight-point Likert scale (ranging from “1 = Exclusively heterosexual” to “8 = No socio-sexual contacts or reactions”).

#### The Sex Role Inventory for College Students (CSRI)

The Sex Role Inventory for College Students (CSRI; Qian et al., 2000) has five categories, including Masculine Positive Category (strong, capable, etc)., Masculine Negative Category (reckless, impatient, etc)., Feminine Positive Category (tender, virtuous, etc)., Feminine Negative Category (lachrymose, hesitating, etc)., and Neutral Category (dedicated, impatient, complacent, etc). Each category is formatted with 20 different personality traits that participants rate themselves based on a five-point Likert scale ranging from “-2 = Totally different” to “+2 = Exactly the same.” Also, the participants could be classified into four sex role types, masculine, feminine, androgynous, and undifferentiated, according to Qian et al. (2000). Its Cronbach’s alpha in the present study was 0.88.

### Preparation of Composite Facial Images

Three composite versions (masculine, average, and feminine) of male and female face stimuli were collected by sexual dimorphism. All the faces (students at Anhui University; 32 males, 32 females) were photographed under standard lighting conditions with neutral facial expression. Also, all of the participants signed a consent form and allowed their photographs to be used in this study and publishing.

To manipulate these photos of faces into our stimuli, we first conducted all the pictures into uniform size (27.09 × 27.09 cm) and the same pixel (1024 × 1024) by PS technology and all the pictures were processed into black and white. Next, we randomly selected 16 male and female faces, respectively, from 64 photographed faces to synthesize male and female average face prototypes by using Fanta Morph 5.9 software. Then, we created the landmark points in each face that identified the shapes, positions, and outlines of these faces. Furthermore, we slightly tweaked the locations of the landmark points in each picture.

To produce an image composed of the two faces, we equated the numeric (pixels) of the landmark points in two pictures. Then, by using the same method, the composite images of the two faces were further synthesized with the other composite images composed of two faces, resulting in an image made of four faces. In this way, we eventually gained two prototype faces that formed of 16 faces.

**FIGURE 1:**
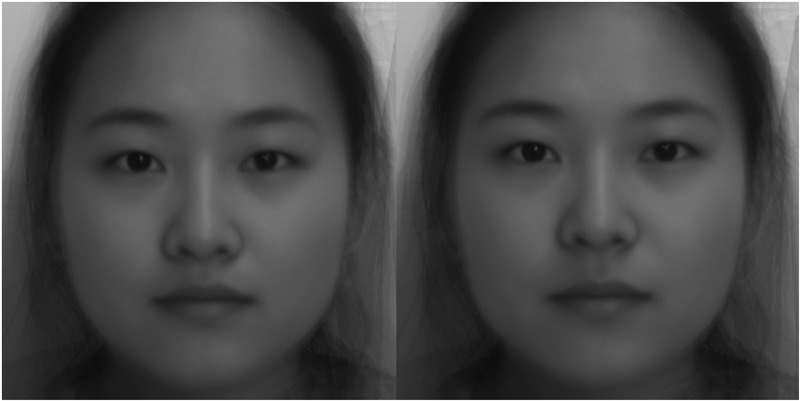
Facial images of a female that were “feminized” and “masculinized” 50% in shape. Left, Chinese female, feminized; right, Chinese female, masculinized.

**FIGURE 2:**
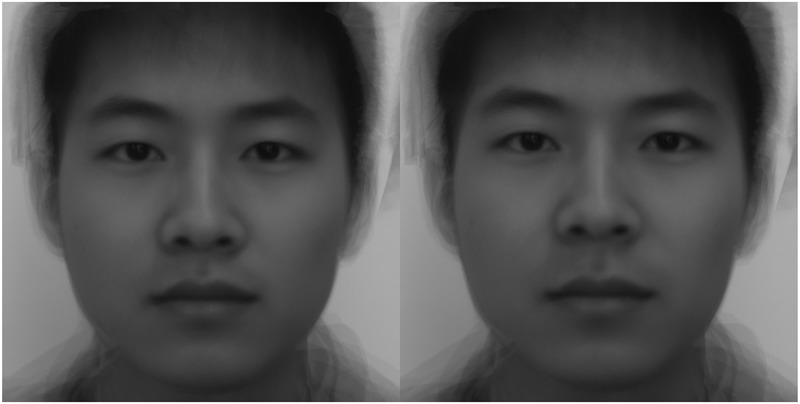
Facial images of a male that were “feminized” and “masculinized” 50% in shape. Left, Chinese male, feminized; right, Chinese male, masculinized.

After that, we manipulated the sexual dimorphism in facial images by using the website https://webmorph.org//, created by DeBruine and others at the School of Psychology (DeBruine, 2017), University of Glasgow. We uploaded the average male and female face prototypes to the website for processing. Lastly, the feminized and masculinized facial stimuli were obtained (see Figure 1, 2). The images of three pairs of female face prototypes were received by the same method.

Then, we randomly selected 40 photographs from the remaining 43 male and 41 female faces, and by manipulating the sexual dimorphism in these facial images, we finally had 46 pairs of masculinized and feminized faces. After that, we invited 80 undergraduates (41 male, 39 female, mean age = 19.12, *SD* = 0.663, ranging from 18 to 21 years) to select which one seemed more masculine (see Table 1). Binomial statistical analysis of the results showed that the masculinization of all the matched control groups was significant. Finally, we randomly selected 20 pairs of faces (half male and half female) as the experimental materials.

**Table 1:** Evaluation of experimental facial stimuli.

Picture number	5	7	8	9	12	13	14	19	20	23	24	25	26	28	37	39	41	42	45	46
Sex	M	F	F	F	M	F	M	M	M	F	F	F	M	M	F	F	M	F	M	M
Masculine selection	78	77	77	80	78	77	78	76	76	79	75	71	75	70	78	79	77	78	74	73
Feminine selection	2	3	3	0	2	3	2	4	4	1	5	9	5	10	2	1	3	2	6	7

### Apparatus

Stimuli were presented on an 18.5-in. monitor at a resolution of 1,024 × 768 pixels and with a refresh rate of 60 Hz. Eye movements were captured and recorded by an EyeLink 1000 Desktop Eye Tracking System (SR Research Ltd., Mississauga, ON, Canada). The system has a sampling rate of 1,000 Hz. The distance between monitor and chin rest was 60 cm. To ensure participants were at ease and to minimize unnecessary head movements, a chin rest was used. The experiment program was created using SR Research Experiment Builder software (version 1.10.165), which is compatible with the EyeLink 1000 eye tracker. Participants viewed the stimuli using both eyes, but only the position of the left eye was tracked and recorded. The eye tracker was calibrated using a series of nine fixed targets distributed around the display, followed by a nine-point accuracy test.

### Procedure

The experiment was conducted in a psychological eye movement laboratory, which is a quiet and undisturbed setting. Psychology students were selected as experimenters and individually tested participants. On arrival, participants read and signed an informed consent form that briefly described the content and procedure of this study. Later, participants were asked to answer the Kinsey Scale. To exclude the bisexuals and asexuals, participants who chose 4 and 8 on the Kinsey Scale were eliminated. After that, participants completed the College Students’ Gender Role Scale for self-evaluation and entered the eye movement experiment.

**FIGURE 3:**
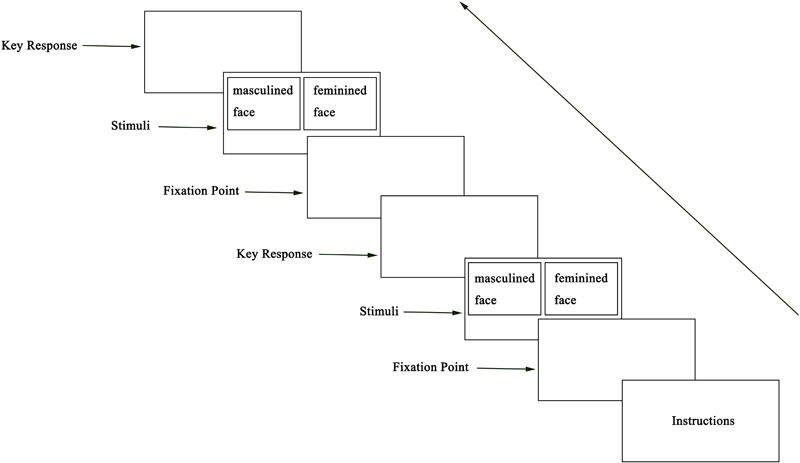
Experiment flowchart.

The eye-tracking session was divided into two stages – (a) Preparation stage: the participant sat in a chair that was 65 cm away from the display device. To ensure the accuracy of experimental data, participants’ heads were fixated, and their lower foreheads were placed on a U-shaped bracket. (b) Experiment stage: The following instructions were presented on the screen – “Hello, welcome to participate in this experiment! This experiment consists of two parts: the calibration part and the experiment part. Please strictly follow the instructions and the hints of the experimenter. Next, we will enter the calibration section. If you are ready, press the Q on the keyboard.” Subsequently, the eye movement instrument was adjusted. The first step consisted of calibrating the eye-tracking system with nine points, and the second step involved validation of errors of the process in which the machine tracked the eyes. After calibration, the screen presented the experimental instruction: “After a while, please look at the gaze points on the screen. Face images will appear on the screen in pairs, which are very similar to each other, with very subtle differences. After they disappear, you need to choose the one which you believe is more attractive. If you think that the left face is more attractive, please press C on the keyboard; if you think the right face is more attractive, please press M on the keyboard. If you have understood the above instructions, please press Y to start.” Twenty-six pairs of male and female faces appeared randomly, each pair consisting of a masculinized and feminized version of the same individual. The order of pairs and the side of the screen on which a given image was shown were both randomized among participants. Participants were instructed to choose which face they thought was more attractive for each pair. The experimental process is shown in Figure 3.

### Data Analysis

Before all analyses, we processed initial eye movement data through the EyeLink Data Viewer analysis software (SR Research). Also, the statistics software package SPSS 16.0 was used for further data analysis. We calculated three eye movement variables: (a) mean number of fixations, which refers to the sum of all fixations in a stimulus; (b) mean first fixation duration, which refers to the average duration (in milliseconds) of the first fixations in a stimulus; and (c) mean pupil size, which refers to the average size (in arbitrary units) of pupil dilation or contraction when viewing stimuli.

The collected data were analyzed in the following ways: first, based on the Kinsey scale, 0–3 were heterosexuals, 5–7 were homosexuals, and 4 (bisexuals), and 8 (asexuals) were excluded from the data. Then, to explore the visual attention patterns of different sexual orientation in watching two different faces (masculinized and feminized), we conducted a 2 (sexual dimorphism: masculine, feminine) × 4 (sexual orientation: homosexual and heterosexual, male and female) mixed analysis of variance (ANOVA). Next, each participant was assigned to four levels (A = masculine, B = feminine, C = androgynous, D = undifferentiated) according to his/her self-rating on the CSRI. To explore the visual attention patterns of participants with different sex roles in watching two different sexual dimorphism stimulations, we conducted a 2 (sexual dimorphism: masculine, feminine) × 4 (sex role: A, B, C, D) mixed ANOVA. Finally, in order to find out the relationship between eye-tracking indicators and the scores of CSRI, we adopted a hierarchical multiple regression analysis.

## Results

The experimental results included behavioral data and eye movement data. The behavioral data were as follows: the probability of choosing masculine faces as more attractive faces in paired faces and the classification of four sex roles. Eye movement data were as follows: the mean number of fixations, the mean duration of the first fixation duration, and the mean pupil size.

In experiment 1, after eliminating the questionnaires with missing data, the data of CSRI were analyzed and excluded from the data of 14 participants. The data of 81 participants (21 homosexual men, 19 heterosexual men, 21 homosexual women, and 20 heterosexual women) were entered into the classification of sex role process. In eye movement data analysis, because of eye fatigue or head movement, the eye movement instrument was unable to record some participants’ data or recorded data inaccurately. Therefore, the data of seven participants were deleted from the eye movement experiment data analysis. Finally, 88 participants (21 homosexual men, 22 heterosexual men, 22 homosexual women, and 23 heterosexual women) entered the eye movement data analysis. Data analysis used SPSS16.0.

At the onset of data analysis, we analyzed if eye moment indicator differences exist within gender. A significant difference was found between gender and the number of fixations (*t* = 2.039, *p* = 0.042, Cohen’s *d* = 0.096). But the results showed no differences between gender and pupil size (*t* = 0.155, *p* = 0.877) as well as the first fixation duration (*t* = 1.758, *p* = 0.079).

### Sexual Orientation and Sexual Dimorphism on Facial Attractiveness

#### The Proportion of Masculinized Faces Chosen as More Attractive Faces

To find the proportion of choosing masculinized faces as more attractive in paired faces, a 2 (gender) × 2 (sexual orientation: homosexuals and heterosexuals) ANOVA was conducted. Data are presented in Table 2. The results indicated that there was a significant main effect of sexual orientation, *F*(1,84) = 6.219, *p* < 0.05, $\eta_{p}^{2}$ = 0.069. Homosexuals (*ME* = 0.504, *SD* = 0.181) have a higher fluency in choosing masculinized faces as more attractive faces in paired faces than heterosexuals (*ME* = 0.414, *SD* = 0.161). Analysis revealed neither a significant main effect of gender, *F*(1,84) = 0.332, *p* = 0.566, $\eta_{p}^{2}$ = 0.004, nor the interaction effect between sexual orientation and gender, *F*(1,84) = 2.968, *p* = 0.089, $\eta_{p}^{2}$ = 0.034.

**Table 2:** The probability of choosing masculine faces as more attractive faces in different types of subjects (M ± SD).

Types	Sexual	M ± SD
	dimorphism	
Homosexual men	Masculinized	0.52 ± 0.14
Heterosexual men	Masculinized	0.37 ± 0.15
Homosexual women	Masculinized	0.48 ± 0.21
Heterosexual women	Masculinized	0.46 ± 0.17

#### Sexual Orientation Difference in Viewing Patterns to Different Sexual Dimorphism Faces

**FIGURE 4:**
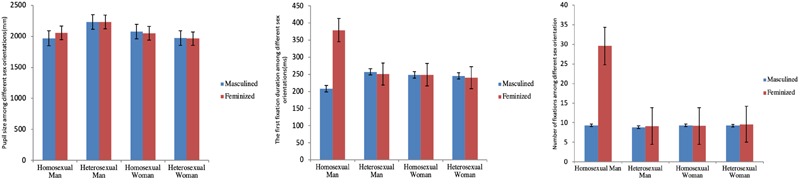
Eye movement indicators among sexual orientations. Left, pupil size among different sexual orientations (mm); middle, the first fixation duration among different sexual orientations (ms); right, number of fixations among different sexual orientations. Error bars represent ± 1 standard error.

A 2 (sexual dimorphism: masculine, feminine) × 4 (sexual orientation: homosexual and heterosexual, male and female) mixed ANOVA was conducted. Means and standard errors for participants gazing at faces are shown in Table 3 and Figure 4.

**Table 3:** Eye movement indicators of subjects with different sexual orientations (M ± SD).

Types	Sexual	Mean pupil size	The first fixation	The number
	dimorphism	(mm)	duration (ms)	of fixations
Homosexual men	Masculinized	1966.38 ± 662.85	207.67 ± 51.87	9.30 ± 1.63
	Feminized	2056.71 ± 533.89	379.27 ± 306.88	29.55 ± 44.98
Heterosexual men	Masculinized	2229.70 ± 554.57	257.29 ± 40.93	8.82 ± 1.52
	Feminized	2234.53 ± 560.45	250.96 ± 38.15	9.12 ± 1.30
Homosexual women	Masculinized	2072.96 ± 467.30	248.08 ± 32.22	9.32 ± 1.32
	Feminized	2049.95 ± 465.15	248.92 ± 35.31	9.15 ± 1.44
Heterosexual women	Masculinized	1971.27 ± 502.60	245.48 ± 48.60	9.27 ± 1.69
	Feminized	1963.13 ± 506.99	239.87 ± 39.40	9.58 ± 1.19

For mean pupil size, analyses did not show a significant effect of group, *F*(3,84) = 1.065, *p* = 0.369, $\eta_{p}^{2}$ = 0.037, or the main effect by sexual dimorphism, *F*(1,84) = 1.104, *p* = 0.296, $\eta_{p}^{2}$ = 0.013. There was a significant interaction effect between sexual dimorphism and subjects’ types, *F*(3,84) = 2.708, *p* < 0.05, $\eta_{p}^{2}$ = 0.088. Simple effect analysis showed that when participants viewed masculine or feminine faces, there was no difference in their pupil size. However, for homosexual men, when viewing masculine faces, their pupil sizes were significantly smaller than when they viewed the feminine faces, *F*(1,20) = 8.409, *p* < 0.01, $\eta_{p}^{2}$ = 0.091.

For the first fixation duration, analyses did not reveal a significant effect of group, *F*(3,84) = 1.937, *p* = 0.130, $\eta_{p}^{2}$ = 0.065. There was a significant main effect of sexual dimorphism, *F*(1,84) = 5.152, *p* < 0.05, $\eta_{p}^{2}$ = 0.058, and a significant interaction effect between sexual dimorphism and sexual orientation, *F*(3,84) = 5.973, *p* < 0.01, $\eta_{p}^{2}$ = 0.176. Simple effect analysis showed that the first fixation duration of homosexual men was significantly shorter than that of heterosexual men (*p* < 0.01), homosexual women (*p* < 0.05), and heterosexual women (*p* < 0.05). When homosexual men were viewing feminine faces, their first fixation duration was significantly longer than that of the other three types (*p* < 0.05). Moreover, for homosexual men, when they were observing masculine faces, their first fixation duration was significantly shorter than their observation of feminine faces, *F*(1,20) = 22.512, *p* < 0.01, $\eta_{p}^{2}$ = 0.211, while for the other three types of subjects, no differences were found.

For the number of fixations, analyses revealed a significant effect of group, *F*(3,84) = 4.382, *p* < 0.01, $\eta_{p}^{2}$ = 0.135, as well as a significant main effect of sexual dimorphism, *F*(1,84) = 5.075, *p* < 0.05, $\eta_{p}^{2}$ = 0.057, and a significant interaction effect between sexual dimorphism and sexual orientation, *F*(3,84) = 4.651, *p* < 0.01, $\eta_{p}^{2}$ = 0.142. Simple effect analysis showed that when participants observed masculine faces in all four types, there was no significant difference in the number of fixations. Conversely, compared to feminine faces, the number of fixations of homosexual men was significantly more than that of the other types (*p* < 0.05). Concerning homosexual men themselves, the number of fixations in viewing masculine faces was significantly less than watching feminine faces, *F*(1,20) = 18.587, *p* < 0.01, $\eta_{p}^{2}$ = 0.181. For the other three types of subjects, no differences were found.

Next, we considered the gender of pictures. Thus, we conducted a 2 (sex of pictures) × 2 (sexual dimorphism) × 4 (sexual orientations) ANOVA. The ANOVA reported a significant main effect of the gender of pictures, *F*(1,84) = 10.48, *p* < 0.005, $\eta_{p}^{2}$ = 0.111. There was a significant interaction effect between the gender of pictures and sexual orientations, *F*(3,84) = 4.424, *p* < 0.005, $\eta_{p}^{2}$ = 0.136, and a significant interaction effect between the gender of pictures and sexual dimorphism, *F*(1,84) = 13.211, *p* < 0.001, $\eta_{p}^{2}$ = 0.136.

### Sex Role and Sexual Dimorphism on Facial Attractiveness

#### Classification of Sex Role

Based on the scores of the two positive scales, each participant was grouped into four sex role types by Spence’s median classification, which calculated the median scores of the Masculine Positive Category (M) and the Feminine Positive Category (F). According to this criterion, the participants were grouped into four types: high M and low F participants were considered a masculine type, low M and high F participants were a feminine type, high M and high F participants were an androgynous type, and low M and low F participants were an undifferentiated type. The results are shown in Table 4.

**Table 4:** Distribution of four sex role types.

	Masculine	Feminine	Androgynous	Undifferentiated	Total
Homosexual men	4 (19.0%)	9 (38.0%)	3 (19.0%)	5 (23.8%)	21
Homosexual women	6 (28.5%)	0 (0%)	10 (47.6%)	5 (23.8%)	21
Heterosexual men	3 (19.0%)	3 (19.0%)	10 (52.3%)	3 (9.5%)	19
Heterosexual women	5 (23.8%)	4 (19.0%)	8 (38.0%)	3 (19.0%)	20
Total	18 (22.6%)	16 (19.0%)	31 (40.4%)	16 (19.0%)	81

We analyzed the frequency of choosing masculinized faces as more attractive faces by ANOVA. The results showed that there was no difference among different sex role types, *F*(3,80) = 0.182, *p* > 0.05. The results are shown in Table 5.

**Table 5:** The probability of choosing masculine faces as more attractive faces in different sex role types of subjects (M ± SD).

Sex role	Sexual	M ± SD
types	dimorphism	
Masculine	Masculinized	0.48 ± 0.18
Feminine	Masculinized	0.44 ± 0.15
Androgynous	Masculinized	0.45 ± 0.17
Undifferentiated	Masculinized	0.47 ± 0.19

#### Sex Role Difference in Viewing Patterns to Different Sexual Dimorphism Faces

**FIGURE 5:**
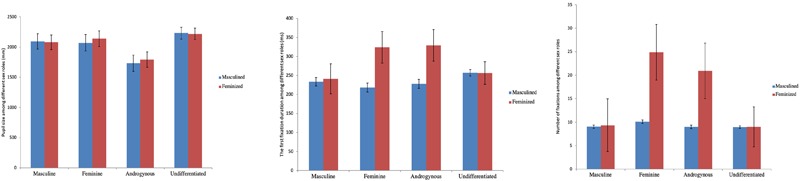
Eye movement indicators among sex roles. Left, pupil size among different sex roles (mm); middle, the first fixation duration among different sex roles (ms); right, number of fixations among different sex roles. Error bars represent ± 1 standard error.

A 2 (sexual dimorphism: masculinized, feminized) × 4 (sex role: masculine, feminine, androgynous, and undifferentiated) mixed ANOVA was conducted. Means and standard errors for participants gazing at faces are shown in Table 6 and Figure 5.

**Table 6:** Eye movement indicators of subjects with different sexual role types (M ± SD).

Types	Sexual	Mean pupil size	The first fixation	The number
	dimorphism	(mm)	duration (ms)	of fixations
Masculine	Masculinized	2094.12 ± 646.80	233.76 ± 29.99	9.04 ± 1.68
	Feminized	2082.21 ± 629.45	241.10 ± 42.39	9.36 ± 1.74
Feminine	Masculinized	2072.62 ± 617.94	218.26 ± 46.43	10.11 ± 1.39
	Feminized	2140.97 ± 526.39	323.88 ± 260.90	24.86 ± 41.91
Androgynous	Masculinized	1731.89 ± 403.70	227.83 ± 50.01	9.00 ± 1.66
	Feminized	1792.69 ± 327.27	329.44 ± 262.89	20.93 ± 33.38
Undifferentiated	Masculinized	2233.59 ± 500.77	257.38 ± 52.80	8.95 ± 1.32
	Feminized	2219.65 ± 510.55	256.32 ± 38.39	9.00 ± 1.04

For mean pupil size, analyses did not show a significant interaction effect, *F*(3,77) = 1.740, *p* = 0.166, or the main effect by sexual dimorphism, *F*(1,77) = 2.227, *p* = 0.140. There was a significant effect by group, *F*(3,77) = 2.787, *p* < 0.05, $\eta_{p}^{2}$ = 0.098. The *post hoc* results showed that when watching masculine faces, the pupil size of the androgynous type was significantly smaller than that of the undifferentiated type (*p* < 0.05). When viewing feminine faces, a marginal difference (*p* = 0.051) was found for the pupil size of androgynous and undifferentiated subjects, and the pupil size of androgynous subjects was smaller than that of undifferentiated subjects.

For the first fixation duration, analyses did not reveal a significant interaction effect, *F*(3,77) = 1.999, *p* = 0.121, or the effect of group, *F*(3,77) = 0.861, *p* = 0.465. There was a significant main effect of sexual dimorphism, *F*(1,77) = 6.400, *p* < 0.05, $\eta_{p}^{2}$ = 0.077. The *post hoc* test showed that when participants were looking at masculine faces, the first fixation duration of feminine subjects was significantly shorter than that of undifferentiated subjects (*p* < 0.05), whereas when they were watching feminine faces, there were no differences among all the four group types. For feminine subjects, their first fixation duration on masculine faces was significantly shorter compared to feminine faces, *F*(1,15) = 5.333, *p* < 0.05, $\eta_{p}^{2}$ = 0.065. Identical results were also found in androgynous subjects, *F*(1,15) = 4.936, *p* < 0.05, $\eta_{p}^{2}$ = 0.060.

For the number of fixations, analyses revealed a significant main effect of sexual dimorphism, *F*(1,77) = 6.346, *p* < 0.05, $\eta_{p}^{2}$ = 0.076. There was no significant interaction effect between sexual dimorphism and subjects’ sex roles, *F*(3,77) = 2.110, *p* = 0.106, or a significant main effect of sex role, *F*(3,77) = 2.390, *p* = 0.075. The *post hoc* test showed that when viewing masculine faces, the number of fixations made by feminine subjects was significantly less than the observation of feminine faces, *F*(1,15) = 6.420, *p* < 0.05, $\eta_{p}^{2}$ = 0.077. Identical results were again found in the androgynous group, *F*(1,15) = 4.203, *p* < 0.05, $\eta_{p}^{2}$ = 0.052.

A 2 (sex of pictures) × 2 (sexual dimorphism) × 4 (sexual orientations) ANOVA was then carried out. The ANOVA reported a significant main effect of the gender of pictures, *F*(1,77) = 10.40, *p* < 0.005, $\eta_{p}^{2}$ = 0.119, and a significant interaction effect between the gender of pictures and sexual orientations, *F*(1,77) = 14.55, *p* < 0.001, $\eta_{p}^{2}$ = 0.159.

### The Effect of Sexual Orientation and Sexual Roles on Eye Movement Indicators

As the 2 × 2 × 4 ANOVA indicated that the interaction effect of sexual orientation and sex role was not significant, we conducted a hierarchical multiple regression analysis in order to consider the effect of sexual orientation and sex role in the first fixation duration and the number of fixations.

The hierarchical multiple regression was conducted with the first fixation duration of participants viewing the masculine faces (the dependent variable). Sexual orientation was entered at stage 1 of the regression and sex role was added at stage 2. The results revealed that at stage 1, sexual orientation contributed significantly to the regression model, *F*(1,79) = 4.361, *p* < 0.05, adjusted *R*^2^ = 0.040, *R*^2^ change = 0.052, β = 0.229. As the variable sex role was entered, it explained an additional 7.4% of the variation in the first fixation duration of watching masculine faces, *F*(2,78) = 4.214, *p* < 0.05. The first fixation duration of participants observing the feminine faces (the dependent variable) was then entered at the sexual orientation, at stage 1, and sex role, at stage 2. Model 1, with the sexual orientation, was the only predictor that explained 7.7% of variance and was considered to be significant, *F*(1,79) = 7.647, *p* < 0.01. Model 2, in which sex role was added, did not explain further variance and was not significant, *F*(1,78) = 0.064, *p* = 0.80, adjusted *R*^2^ = 0.066, *R*^2^ change = 0.001. Furthermore, the number of fixation of participants when observing the feminine face was used as the dependent variable. The independent variable at stage 1 was the sexual orientation. Sex role was put in at stage 2. The results at stage 1 indicated that sexual orientation contributed significantly, *F*(1,79) = 7.020, *p* < 0.05, adjusted *R*^2^ = 0.070, *R*^2^ change = 0.082, β = -0.286. At stage 2, sex role was not significant, *F*(1,78) = 0.120, *p* = 0.730, adjusted *R*^2^ = 0.060, *R*^2^ change = 0.001. Generally speaking, the prediction and function of sexual orientation are more significant than the prediction of sex role.

## Discussion

### General Discussion

Using an eye-tracking task, the present study investigated whether preference differences exist in subjects with different sexual orientations and sex roles from different sexual dimorphism stimuli. Findings from the Bailey et al. (1997) study revealed that compared to heterosexual men, homosexual men preferred masculinized faces, while no significant differences were found between homosexual women and heterosexual women. Most gay males prefer partners who described themselves as masculine (Valentova et al., 2014). In our study, we replicated this finding. Homosexual subjects had a higher fluency in choosing masculine faces as more attractive faces in paired faces than heterosexual groups. In particular, we certainly found that masculine faces are more attractive to homosexual men than heterosexual men. However, no significant differences existed in heterosexual and homosexual female groups. Hence, this supported our hypothesis 1.

Furthermore, Bailey et al. (1997) hypothesized that homosexual men might seek and have similar face preferences to heterosexual women, whereas homosexual women may have similar face preferences to heterosexual men. According to the CSRI questionnaire, the current findings show little evidence of homosexual men treating themselves into a feminine group, and homosexual women treating themselves into a masculine group, hence potentially supporting our hypothesis 2.

Moreover, from findings in sex roles, we also found that compared to feminine faces, masculine faces are more attractive to subjects within feminine type and androgynous type. This partly supported our hypothesis 3, that masculinized faces are more attractive to participants within feminine sex roles. However, no significant differences were detected in the masculine group.

### Sexual Orientation and Sex Role Difference in Viewing Patterns

Pupil size reflects emotional arousal and autonomic activation during affective picture viewing (Partala and Surakka, 2003; Laeng and Falkenberg, 2007; Han and Yan, 2010), and increasing mental load and difficulty of tasks can lead to enlargement of pupil dilation in cognitive tasks (Zhou and Liu, 2009). When viewing masculinized and feminized faces, the average pupil size of the subjects with different sexual orientations was different.

The first fixation duration reflects the early recognition process as well as the sensitivity to materials (Ding et al., 2007). We found that the first fixation duration of homosexual men was significantly shorter than the other three types when viewing masculinized faces, and the first fixation duration of homosexual men was significantly longer than the other three groups when viewing feminine faces. This finding could be explained as in the early recognition process of face images, the sensitivity to the difficulty of processing feminized faces is higher than that of masculinized faces. Further, it also proves that subjects for masculine faces will have a higher level of processing and load. In other words, compared to feminized faces, homosexual men would first take notice of masculinized faces.

The number of fixations reflects the ability of the subjects to deal with stimuli as well as the difficulty of the stimulations (Wen and Zuo, 2012). In our study, we found that the number of fixations of homosexual male watched feminized faces significantly more than masculinized faces. The number of fixations reflected the difficulty of processing, indicating that compared to masculinized faces, homosexual men judged feminized faces as more difficult and consequently processed more deeply. This result is similar to research of Sulikowski et al. (2013) where homosexual men showed less sensitivity than heterosexual men in terms of attractiveness to female faces. The results were consistent with the data of pupil dilation and first fixation duration, suggesting that sexual orientation was one of the factors affecting face attractiveness.

Our study further revealed that when watching masculinized faces, the pupil size of androgynous subjects was significantly smaller than that of undifferentiated subjects. Moreover, when viewing masculine faces, the first fixation duration of participants in the feminine group was significantly shorter than that in the undifferentiated group. Moreover, compared to the viewing of feminine faces, the first fixation duration and the number of fixations of the feminine and the androgynous group were shorter. These results may indicate that sex role type is one of the factors influencing the face attractiveness of different sexual orientation subjects.

### The Feminization and Masculinization Bias of Homosexuals

Besides, we speculated that under the condition of dividing participants by their sexual orientation, the proportion of sex roles would change compared to Qian et al. (2000). In our study, 40.4% of the subjects were found to be undifferentiated. This may relate to the average age of the participants. We mainly studied college students, aged between 18 and 24 years old; at this age, young people are mostly in the “psychological weaning period.” Their concept of psychological gender is not perfect enough to support them to affirm their sex role. After classifying homosexual men according to their sexual orientation, we found that most of the male homosexual’s sex role was a feminine type, but no female homosexual’s sex role types were feminine. This is partly consistent with the findings of Zheng and Zheng (2009); that is, homosexual men have a feminized inclination to a certain extent. We believe that this may be related to the sex role type of subjects. Chinese gay men mostly use “1,” “0,” and “both” to distinguish their identity in their partnership, which is equal to “tops,” “bottoms,” and “versatile.” In the social and cultural context, “1” is given more meaning (i.e., virile, strong, and so forth), and “0” is considered to be a sissy or womanish (Bai et al., 2013). Previous studies found that compared to the masculinized male faces, the “1” prefer feminized male faces, and the “0” prefer partners with masculinized faces (Zheng and Zheng, 2015; Zhang et al., 2018). Therefore, we suggest that the feminization bias of homosexual men may be partly due to the differences in individual identities in their partnerships.

### Sexual Orientation and Sex Role

The data of regression analysis suggested that the predicted function of sexual orientation is more significant than the prediction of sex role. This is consistent with the previous study, which proposed that the preference for faces may differ between heterosexuals and homosexuals (Glassenberg et al., 2010). Further, sexual orientation plays an important role in judgment (Ishai, 2007; Lippa, 2007), which could highlight that it is a predicted function in this study. However, considering the sex role, the current study was not powerful enough to prove the significance of facial attractiveness. Hence, the research of sex role still needs to be carried out.

### Limitations and Future Directions

This study is a preliminary exploration of different sexual orientations and sex role preferences in facial attractiveness. However, several limitations of this study should be taken into consideration when making future comparisons.

Initially, it is necessary to increase the sample size since the power is directly based on it. Meanwhile, the participants selected in this study are college students, whose sample representativeness has certain limitations. Thus, in future studies, we not only need to expand the sample size but also further investigate the different ages and occupations of homosexuals and heterosexuals.

Secondly, this study did not put the menstrual cycle into consideration. Several studies have found that the menstrual cycle may have effects on women’s judgements for various traits (Penton-Voak et al., 1999; Little et al., 2007; Jones et al., 2008; Little and Jones, 2012); besides, Roberts et al. (2004) have discovered that female faces were more attractive at peak fertility in the menstrual cycle. Therefore, it is recommended for future studies to classify the influence of menstrual cycle.

Third, the role of homosexuals in their partnership should also be considered. Thus, in future studies, we can recruit subjects according to their roles in a homosexual partnership. Lastly, in terms of the evaluation of indicators, we can further combine functional magnetic resonance imaging (fMRI), event-related potential (ERP), and other cognitive neuroscience and technology equipment to provide more objective and scientific indicators for face preference.

## Ethics Statement

The study was approved by the Human Research Ethics Committee of Anhui University. All participants gave consent to participate in the study and principles expressed in the Declaration of Helsinki were closely followed. Participants were undergraduate students. Informed consent was obtained in written form from all participants. The youngest participant was 18 years old. We did not obtain informed consent from the next of kin, caretakers, or guardians on behalf of the minors/children enrolled in our study. These college students were considered to have comparable intelligence and ability, and able to take charge of their behaviors.

## Author Contributions

JH, QC, LS, and XJ conceived and designed the experiments. QC, LS, and XJ performed the experiments. QC and XJ analyzed the data. QC, LS, and CH contributed to the materials and analysis tools. JH, QC, XJ, LS, and CH wrote the manuscript. JH, QC, and LS discussed the result. JH, QC, and CH gave the final approval of the version to be published.

## Conflict of Interest Statement

The authors declare that the research was conducted in the absence of any commercial or financial relationships that could be construed as a potential conflict of interest.
